# Refracture and mortality following hospitalization for severe osteoporotic fractures: The Fractos Study

**DOI:** 10.1002/jbm4.10507

**Published:** 2021-05-14

**Authors:** Christian Roux, Thierry Thomas, Julien Paccou, Geoffray Bizouard, Anne Crochard, Emese Toth, Magali Lemaitre, Frédérique Maurel, Laure Perrin, Florence Tubach

**Affiliations:** ^1^ Department of Rheumatology Cochin Hospital, Assistance Publique ‐ Hôpitaux de Paris Centre, Institut National de la Santé et de la Recherche Médicale (INSERM) Unités Mixtes de Recherche (UMR) 1153, Université de Paris Paris France; ^2^ Department of Rheumatology Hôpital Nord, Centre Hospitalier Universitaire (CHU) Saint‐Etienne, INSERM U1059, Lyon University Saint‐Etienne France; ^3^ Department of Rheumatology Lille University, CHU Lille, Marrow Adiposity and Bone Laboratory (MABlab) ULR 4490 Lille France; ^4^ IQVIA Courbevoie France; ^5^ UCB Pharma Colombes France; ^6^ Department of Public Health, Pharmacoepidemiology Center (Cephepi) Sorbonne University, INSERM, Institut Pierre Louis d'Epidémiologie et de Santé Publique, AP‐HP Sorbonne University, Pitié‐Salpêtrière Hospital, CIC‐1422 Paris France

**Keywords:** OSTEOPOROSIS, GENERAL POPULATION STUDIES, FRACTURE RISK ASSESSMENT, FRACTURE PREVENTION, THERAPEUTICS

## Abstract

Severe osteoporotic fractures (hip, proximal humerus, pelvic, vertebral and multiple rib fractures) carry an increased risk of mortality. This retrospective cohort study in the French national healthcare database aimed to estimate refracture and mortality rates after severe osteoporotic fractures at different sites, and to identify mortality‐related variables. A total of 356,895 patients hospitalized for severe osteoporotic fracture between 2009 and 2014 inclusive were analyzed. The cohort was followed for 2 to 8 years up to the study end or until the patient died. Data were extracted on subsequent hospitalizations, refracture events, treatments, comorbidities of interest and survival. Time to refracture and survival were described using Kaplan‐Meier analysis by site of fracture and overall. Mortality risk factors were identified using a Cox model. Hip fractures accounted for 60.4% of the sample (*N* = 215,672). In the 12 months following fracture, 58,220 patients (16.7%) received a specific osteoporosis treatment, of whom 21,228 were previously treatment‐naïve. The 12‐month refracture rate was 6.3% (95% confidence interval [CI], 6.2%–6.3%), ranging from 4.0% (95% CI, 3.7%–4.3%) for multiple rib fractures to 7.8% (95% CI, 7.5%–8.1%) for pelvic fractures. Twelve‐month all‐cause mortality was 12.8% (95% CI, 12.7%–12.9%), ranging from 5.0% (95% CI, 4.7%–5.2%) for vertebral fractures to 16.6% (95% CI, 16.4%–16.7%) for hip fractures. Osteoporosis‐related mortality risk factors included fracture site, previous osteoporotic fracture (hazard ratio 1.21; 95% CI, 1.18–1.23), hip refracture (1.74; 95% CI, 1.71–1.77), and no prior osteoporosis treatment (1.24; 95% CI, 1.22–1.26). Comorbid cancer (3.15; 95% CI, 3.09–3.21) and liver disease (2.54; 95% CI, 2.40–2.68) were also strongly associated with mortality. In conclusion, severe osteoporotic fractures, including certain non‐hip nonvertebral fractures, carry a high burden in terms of mortality and refracture risk. However, most patients received no anti‐osteoporotic treatment. The findings emphasize the importance of better management of patients with severe fractures, and of developing effective strategies to reduce fracture risk in patients with osteoporosis. © 2021 The Authors. *JBMR Plus* published by Wiley Periodicals LLC on behalf of American Society for Bone and Mineral Research.

## 1 INTRODUCTION

Osteoporotic fractures are a major source of disability, loss of autonomy and reduced quality of life.^(^ ^1^, ^2^, ^3^, ^4^, ^5^, ^6^ ^)^ Two major epidemiological features of osteoporosis highlight the view that this disease is becoming an important threat to the elderly population and generate an even heavier burden to health care. First, the number of frail elderly patients who are at high risk of falls and fractures is expected to increase dramatically in the next years and decades. It is now well demonstrated that fractures at certain locations, notably the vertebrae^(^ ^7^ ^)^ and the hip,^(^ ^4^ ^)^ carry an increased risk of mortality.^(^ ^4^, ^8^, ^9^, ^10^ ^)^ However, this is also the case for other fracture sites such as the pelvis and the proximal humerus,^(^ ^9^ ^)^ for which much less information is available. Part of this increased mortality risk is related to refractures,^(^ ^11^ ^)^ whereas the main risk factor of incident fracture is having a history of fracture. Second, although the average risk of sustaining a fracture is twofold higher in patients with prevalent fractures,^(^ ^12^ ^)^ there is a growing body of evidence that fractures cluster in time, with a particularly high risk of refracture in the 2 to 3 years following a fracture, decreasing thereafter. This temporary increase defines the imminent fracture risk,^(^ ^13^ ^)^ which can have implications for patient management. During this high‐risk period, osteoporosis has a major impact on refracture, utility loss and mortality,^(^ ^14^, ^15^, ^16^, ^17^, ^18^ ^)^ depending on features such as age,^(^ ^14^ ^)^ comorbidities and the location of the fracture.^(^ ^19^ ^)^

For these reasons a number of international^(^ ^20^ ^)^ and national^(^ ^21^ ^)^ guidelines are available, to select patients with a high risk of fracture, or refracture, who are at the highest priority for receiving treatment. Although there is no reason for not following these recommendations, at least in high‐income countries with universal healthcare coverage, a wide treatment gap exists between recommended and actual practice.^(^ ^22^ ^)^ With this in mind, we have performed a cohort study in the French national healthcare data base. The principal objective was to assess the short‐term consequences of severe osteoporotic fractures at different sites in terms of refracture and mortality. Secondary objectives were to identify risk factors associated with mortality and to describe treatment patterns.

## 2 PATIENTS AND METHODS

The FRACTOS study was a retrospective cohort study performed using the French National Health database (Système National des Données de Santé [SNDS]). The cohort was composed of all patients hospitalized for severe osteoporotic fracture between January 1, 2009 and December 31, 2014.

For the purposes of this study, “severe osteoporotic fractures” covered fractures of the hip, proximal humerus, pelvis, and thoracic or lumbar vertebrae, as well as multiple rib fractures. These fractures carry an elevated mortality risk.^(^ ^4^, ^21^ ^)^ and are the fracture sites for which the French guidelines and health authorities recommend specific osteoporosis treatments. For this reason, individuals with fractures at any of these sites should receive the same quality of care. It should be noted that the present definition of “severe osteoporotic fractures” is not identical as that proposed by the International Osteoporosis Foundation for “major osteoporotic fractures” which also includes distal forearm fractures, but excludes pelvic and rib fractures.^(^ ^23^ ^)^

The first hospitalization for osteoporotic fracture during this period was considered the index event. The cohort was followed prospectively for 2 to 8 years after the index event up to the study end on December 31, 2016 (or until the patient died). This was the cutoff date for which exhaustive finalized data were available in the SNDS when the study was initiated. In addition, historical data on previous fractures, comorbidities and treatments were retrieved from the date of availability of the database (January 1, 2006) until the index event. Over the follow‐up period, data were extracted concerning subsequent hospitalizations, refracture events, treatments, comorbidities of interest, and survival.

### 2.1 Data source

The SNDS database is the repository of healthcare data of all individuals insured by the French national health insurance system,^(^ ^24^, ^25^ ^)^ made up of several regimens according to the professional occupation of the insurees. The largest of these regimes is the General Regimen, which accounts for 88% of the French population, and was the basis of this study.

The SNDS database contains comprehensive data on healthcare resource consumption by all insurees since January 1, 2006 for hospitalizations and since January 1, 2008 for community healthcare delivery. These include data on hospitalizations (both overnight and day hospitalization), in the form of hospital discharge summaries for all individual stays with information on the reason for hospitalization, coded using the International Classification of Diseases and Related Health Problems, 10th Revision (ICD‐10) classification. Procedures performed in hospital are documented, although no information is available on the results of any tests or on clinical decision‐making. Information on medication delivered in hospital is generally not available, with the exception of a number of selected and listed expensive treatments. Full information is available for reimbursed healthcare consumption in the community, notably physician consultations, medication delivered in pharmacies and all tests (including laboratory tests and imaging). Insurees qualifying for, and receiving, full healthcare reimbursement either because they have a listed serious chronic disease (ALD status) or because they have low incomes (CMU status) are identified. The date, but not the cause, of death is documented for all insurees when they die.

### 2.2 Participants

Patients aged ≥50 years insured by the General Regimen of the French national healthcare insurance and hospitalized for a severe osteoporotic fracture, identified from diagnostic codes in the hospital discharge summary, during the inclusion period were eligible.

Patients who changed their insurance regimen during the study period (from the index event until the end of the study) were not eligible since exhaustive documentation of outcomes of interest throughout the period could not be guaranteed. Patients with a history of Paget's disease, cancer, infectious arthritis, or bone fragility secondary to malignant disease or to surgical interventions documented in the SNDS database in the 3 years prior to the index fracture event were not eligible. A complete listing of the ICD‐10 codes used to assess the eligibility criteria is provided in Supplemental Table S1.

### 2.3 Fracture events

Severe osteoporotic fractures were identified by diagnostic codes in the hospital discharge summary according to the ICD‐10 disease classification (Supplemental Table S1). Open fractures, considered as likely to be of traumatic origin, were identified by the last digit in the ICD‐10 code and these were excluded from the definition, as were hospitalizations with identified traumatic injury, or with fractures associated with polytrauma (other than multiple rib fractures). Hospitalizations with procedure codes for care of an existing prosthesis were also excluded. In addition, hospitalizations for multiple fractures and those with “osteoporosis with current pathological fracture” (ICD‐10 code M80) on the hospital discharge summary were not considered either, since a unique fracture site was not identifiable for these hospitalizations.

Refractures were documented from hospital discharge summaries, as described in the previous paragraph for the index fracture. Refractures were defined as any fracture event after the index fracture hospitalization, either occurring at a different site, or occurring at the same site ≥60 days after the index fracture. All hospitalizations for osteoporotic fractures were included, regardless of site, including severe fracture sites as defined above, as well as other single osteoporotic fractures at other sites, including the distal femur, tibia, wrist, or forearm.

### 2.4 Primary variables extracted from the SNDS database

Gender and age of patients at the time of the index fracture event were extracted. Comorbidities present at the index date (Supplemental Table S2) were identified using diagnostic proxies relying on hospital discharge summaries, ALD status or drug delivery over the year preceding the index hospitalization.^(^ ^26^ ^)^

The site of the index fracture was documented. Osteoporotic fracture history in the 3 years prior to the index fracture event were identified using the same definitions as for the index hospitalization. Delivery of specific antiosteoporotic drugs in the 2 years before the index fracture event and subsequent delivery at any time after the index event were identified. These included all treatments available during the study period, namely bisphosphonates, denosumab, raloxifene, strontium ranelate, teriparatide, and hormone substitution therapy. The dates of first and last delivery of treatment were documented. Calcium and vitamin D supplementation were not considered as specific antiosteoporotic drugs.

A number of potential refracture risk factors, were identified. These include sociodemographic variables (age at index fracture and gender), osteoporosis‐related variables (site of index fracture, fracture history, and osteoporosis treatment history), and comorbidities present at the index date. Certain prespecified medical conditions and treatments documented for the first time in the database after the index fracture event were identified, namely cancer, stroke or hemiplegia, Parkinson's disease, and initiation of corticosteroid treatment.

### 2.5 Derived variables

The Charlson comorbidity index at the index date was calculated as recommended for the SNDS database.^(^ ^26^ ^)^

Osteoporotic treatment duration was determined as the time between the first documented delivery of medication and the end of the theoretical treatment period covered by the last delivery (or until the end of the study or until the patient died) without treatment interruption. A treatment was considered to be interrupted if the interval between the theoretical end of a treatment and the next treatment dispensation was >90 days. Treatment provided following the index fracture was then classed as initiation (no treatment in the 2 years preceding the index fracture and first delivery documented during the follow‐up period), discontinuation (last delivery documented during the follow‐up period), continuous (deliveries continuing without interruption) and restarted (delivery of a treatment following a period of interruption that included the index date). Switches between specific osteoporosis treatments were not considered as discontinuation events. Persistence with treatments taken after the fracture was evaluated from the time the treatment was started using Kaplan‐Meier survival analysis.

### 2.6 Outcomes

Two outcomes were evaluated, namely refracture and death. Time to refracture was defined as time from index fracture to subsequent hospitalization for osteoporotic fracture ≥60 days after the index fracture. For patients with multiple refracture events, the first hospitalization after the index fracture was considered. Survival was defined as the time from index fracture to death. For patients who died, variables associated with mortality were evaluated, with the specific goal of assessing whether refracture events were associated with increased mortality.

### 2.7 Statistical analysis

Two study populations were of interest. The *analysis population* consisted of all patients fulfilling the eligibility criteria and the *follow‐up population* consisted of all members of the analysis population with at least 1 day of follow‐up after the index hospitalization. Presentation of the characteristics of patients in the analysis population and of their fractures is descriptive.

Time to refracture and survival were described using the Kaplan‐Meier method, firstly in the whole follow‐up population, and secondly by site of the index fracture. Mortality risk factors were identified using a Cox proportional hazard model. The risk factors considered are reported in Supplemental Table S2. Incident cancer, Parkinson's disease and stroke, corticosteroid use initiated after the index fracture, and refracture were considered as time‐dependent variables. A stepwise model was implemented, using backward selection with *p* < 0.05 as removal criterion. Patients with incident Paget's disease, infectious arthropathy, or secondary osteoporosis identified after the index hospitalization were censored at the date these conditions were identified.

Age‐ and gender‐standardized mortality rates (SMR) in the year following the index fracture were calculated for each fracture type using general population mortality data from the French national statistics office^(^ ^27^ ^)^ as the reference.

All analyses were performed using SAS software, version 16.2 (SAS Institute, Inc., Cary, NC, USA).

### 2.8 Ethics

The study was conducted in accordance with all relevant regulatory requirements. Use of the SNDS database is regulated by the National Health Data Agency (Institut National des Données de Santé). The FRACTOS study was authorized by the CEREES (the French expert ethical committee for health technonogy studies and evaluations) in February 2018 and by the French national data protection agency (Commission Nationale de l'Informatique et des Libertés) in March 2018.

## 3 RESULTS

### 3.1 Study population

Overall, 560,499 patients with at least one hospitalization for severe osteoporotic fracture were identified, corresponding to 93,000 individuals on average hospitalized each year in France. This translates into a crude incidence rate of ~1.4 cases/1000 in the general population and ~3.6 cases /1000 in the population ≥50 years of age.

Of these 560,499 patients, 356,895 (63.7%) fulfilled the eligibility criteria and 347,784 had at least 1 day of follow‐up after the index hospitalization. The median follow‐up duration was 39.1 months (interquartile range: 21.8–60.5); 277,842 patients (82.2%) had a follow‐up duration of at least 2 years, and 185,039 (51.9%) were followed until the end of the study period, including 136,929 (38.4%) with a follow‐up duration of at least 5 years. A patient flow diagram is presented in Supplemental Figure S1.

The characteristics of the analysis population at the index hospitalization are presented in Table 1. The same variables are presented by gender in Supplemental Table S3. Hip fractures were the most frequent severe osteoporotic fractures encountered. However, fractures at sites other than the hip and vertebrae accounted for over 30% of all severe osteoporotic fractures. The distribution of fractures differed between men and women, with vertebral and multiple rib fractures being more frequent in men than in women, and hip and pelvic fractures being more frequent in women. Overall, 4.0% of patients had been previously hospitalized for a fracture in the 3 years preceding the index hospitalization (2.2% of men and 4.6% of women; Supplemental Table S3). The mean age of the analysis population was 79 years, patients with hip or pelvis fractures being older than those with fractures at other sites, and the mean age being lower in men than in women at all fracture sites. Three‐quarters of the patients were women. However, multiple rib fractures most frequently occurred in men and 40.4% of patients hospitalized for vertebral fractures were men. Diabetes and chronic lung disease were the most frequent comorbidities.

**TABLE 1:** Characteristics of patients at the index hospitalization by fracture type (analysis population: period January 1, 2009–December 31, 2014)

Characteristic	Hip	Vertebra	Pelvis	Multiple ribs	Proximal humerus	Total
Patients (% of total), *n* (%)	215,672 (60.4)	32,231 (9.0)	38,620 (10.8)	17,450 (4.9)	52,922 (14.8)	356,895 (100)
Fracture within 3 previous years, *n* (%)	9286 (4.3)	838 (2.6)	1725 (19.5)	448 (2.6)	1912 (3.4)	14,209 (4.0)
Age (years)						
Mean ± SD	81.8 ± 10.6	70.5 ± 12.4	79.5 ± 11.8	71.9 ± 13.3	73.8 ± 12.1	78.8 ± 12.0
≤65 years, *n* (%)	20,623 (9.6)	12,042 (37.4)	5,779 (15.0)	6255 (35.8)	14,309 (27.0)	59,008 (16.5)
65–80 years, *n* (%)	47,470 (22.0)	10,859 (33.7)	9,243 (23.9)	4973 (28.5)	18,244 (34.5)	90,789 (25.4)
>80 years, *n* (%)	147,579 (68.4)	9,330 (28.9)	23,598 (61.1)	6222 (35.7)	20,369 (38.5)	207,098 (58.0)
Gender, *n* (% women)	167,431 (77.6)	19,221(59.6)	29,767 (77.1)	7626 (43.7)	41,713 (78.8)	265,758 (74.5)
Charlson score						
Mean ± SD	0.6 ± 1.0	0.4 ± 0.9	0.5 ± 1.0	0.5 ± 1.0	0.5 ± 0.9	0.6 ± 1.0
0, *n* (%)	130,917 (60.7)	22,730 (70.5)	24,809 (64.2)	11,183 (64.1)	35,242 (66.6)	224,881 (63.0)
1–2, *n* (%)	74,021 (34.3)	8609 (26.7)	12,199 (31.6)	5565 (31.9)	16,035 (30.3)	116,429 (32.6)
3–4, *n* (%)	7652 (3.5)	615 (1.9)	1139 (2.9)	497 (2.8)	1149 (2.2)	11,052 (3.1)
≥5, *n* (%)	3082 (1.4)	277 (0.9)	473 (1.2)	205 (1.2)	496 (0.9)	4533 (1.3)
Comorbidities, *n* (%)^a^						
Diabetes	23,950 (11.1)	3700 (11.5)	4532 (11.7)	2411 (13.8)	7547 (14.3)	42,140 (11.8)
CLD	24,452 (11.3)	3766 (11.7)	4695 (12.2)	2699 (15.5)	6092 (11.5)	41,704 (11.7)
Dementia	29,362 (13.6)	1203 (3.7)	3105 (8.0)	874 (5.0)	3025 (5.7)	37,569 (10.5)
Stroke	7432 (3.4)	595 (1.8)	948 (2.5)	386 (2.2)	1052 (2.0)	10,413 (2.9)
CHF	9825 (4.6)	697 (2.2)	1583 (4.1)	568 (3.3)	1121 (2.1)	13,794 (3.9)
MI	3494 (1.6)	309 (1.0)	521 (1.3)	227 (1.3)	486 (0.9)	5037 (1.4)

Abbreviations: CHF, congestive heart failure; CLD, chronic lung disease; MI, myocardial infarction; SD, standard deviation.

^a^ Only comorbidities used to construct the Charlson comorbidity index and identified in >1% of patients overall are listed.

### 3.2 Specific osteoporosis treatments

In the 2 years before the index fracture, 59,286 patients (17.0%) had been delivered a specific osteoporosis treatment at least once and 8.4% were under treatment at the time of the fracture (Table 2). Treatments are presented by gender in Supplemental Table S4. In men, these proportions were 3.2% and 4.3%, respectively, whereas in women 21.7% of women had received a specific osteoporosis treatment at least once before the index fracture. The proportion of patients receiving such a treatment before the index fracture was lowest for multiple rib fractures and highest for fractures of the pelvis (Table 2).

**TABLE 2:** Specific antiosteoporotic drug treatments

Follow‐up population	Hip (*n* = 208,102)	Vertebra (*n* = 31,979)	Pelvis (*n* = 38,051)	Multiple ribs (*n* = 17,184)	Proximal humerus (*n* = 52,468)	Total (*n* = 347,784)
Before index fracture, *n* (%)						
At least one delivery	32,930 (15.8)	6125 (19.2)	9270 (24.4)	2200 (12.8)	8761 (16.7)	59,286 (17.0)
At time of index fracture	15,273 (7.3)	3390 (10.6)	5056 (13.3)	1203 (7.0)	4450 (8.5)	29,372 (8.4)
During 12 months after index fracture, *n* (%)						
At least one delivery	31,385 (15.1)	8250 (25.8)	8683 (22.8)	1775 (10.3)	8127 (15.5)	58,220, (16.7)
Treatment continued^a^	15,273 (7.3)	3390 (10.6)	5056 (13.3)	1203 (7.0)	4450 (8.5)	29,372 (8.4)
Treatment restarted^b^	4048 (1.9)	1112 (3.5)	1166 (3.1)	196 (1.1)	1098 (2.1)	7620 (2.2)
Treatment initiated^c^	12,064 (5.8)	3748 (11.7)	2461 (6.5)	376 (2.2)	2579 (4.9)	21,228 (6.1)

^a^ Treatment continued: treatment ongoing at time of index fracture and delivery continuing without interruption thereafter.

^b^ Treatment restarted: delivery of a previous treatment after the index fracture, following a period of interruption.

^c^ Treatment initiation (no treatment in the 2 years preceding the index fracture and first delivery documented after the index fracture.

Following the index fracture event, 71,913 patients were delivered a specific osteoporosis treatment at least once (20.7%), including 58,220 (16.7%) who received treatment within 12 months of the fracture. A large gender difference in treatment rates was observed, with 20.8% of women receiving a treatment, compared to 4.6% of men. For patients with vertebral fractures, the proportion of patients treated increased from 19.2% before the index fracture to 25.8% afterward. No such increase was observed for the other fracture sites (Table 2). In the 12 months following the index fracture, 6.1% of previously treatment‐naïve patients initiated a specific osteoporosis treatment for the first time. This proportion was 5.8% in patients with index hip fractures and 11.7% in those with index vertebral fractures (Table 2). The median interval between the index hospitalization and treatment initiation was 6.3 months (interquartile range: 2.3–17.7 months). For treatments ongoing at the time of the index fracture or initiated thereafter, treatment persistence following the index fracture was 49.0% at 12 months, 31.7% at 2 years, and 12.9% at 3 years. In addition to these specific osteoporosis treatments, 74,858 previously treatment‐naïve patients (86.4% of treatment‐naïve patients) were delivered a prescription for calcium or vitamin D after the index fracture.

### 3.3 Refracture

Overall, 55,831 patients (16.1%) experienced at least one refracture leading to hospitalization during the follow‐up period; this concerned 10.2% of men and 17.9% of women (Supplemental Table S4). The rate of refracture at 12 and 36 months was lowest for index multiple rib fractures (4.0% and 9.6%, respectively) and highest for index fractures of the pelvis (7.8% and 18.0%, respectively) (Table 3). For those patients experiencing a refracture, the median duration between the index fracture and refracture was 19 months. Kaplan‐Meier survival curves for time to refracture are presented by index fracture site in Supplemental Figure S2. More than one refracture over the follow‐up period were observed for 8302 patients (2.4%).

**TABLE 3:** Refracture rates

	Site of index fracture					
Parameter	Hip (*n* = 208,102)	Vertebra (*n* = 31,979)	Pelvis (*n* = 38,051)	Multiple ribs (*n* = 17,184)	Proximal humerus (*n* = 52,468)	Total (*n* = 347,784)
Refracture (*n*)	34,039	4372	7440	1948	8032	55,831
Refracture rate at 12 months, % (95% CI)	6.6 (6.5–6.7)	5.5 (5.3–5.8)	7.8 (7.5–8.1)	4.0 (3.7–4.3)	5.1 (4.9–5.3)	6.3 (6.2–6.3)
Refracture rate at 24 months, % (95% CI)	11.7 (11.6–11.9)	9.0 (8.8–9.3)	13.3 (12.9–12.7)	7.1 (6.7–7.5)	9.0 (8.8–9.3)	10.9 (10.8–11.1)
Refracture rate at 36 months, % (95% CI)	16.1 (15.9–16.3)	11.6 (11.2–11.9)	18.0 (17.6–18.4)	9.6 (9.1–10.0)	12.5 (12.2–12.8)	14.9 (14.7–15.0)
Time from index fracture to refracture (months)^a^						
Mean ± SD	23.7 ± 19.6	23.0 ± 20.1	23.0 ± 19.5	24.8 ± 20.1	26.1 ± 20.6	24.0 ± 19.8
Median [IQR]	18.7 [8–35]	17.4 [6–35]	18.0 [7–34]	20.0 [8–37]	21.5 [9–39]	19.0 [8–36]
Site of first refracture, *n* (%)						
Hip	16,794 (49.3)	1,453 (33.2)	3,661 (49.2)	783 (40.2)	3,814 (47.5)	26,505 (47.5)
Vertebra	950 (2.8)	804 (18.4)	429 (5.8)	155 (8.0)	341 (4.2)	2,679 (4.8)
Pelvis	3,305 (9.7)	465 (10.6)	871 (11.7)	232 (11.9)	638 (7.9)	5,511 (9.9)
Multiple ribs	519 (1.5)	134 (3.1)	206 (2.8)	151 (7.8)	196 (2.4)	1,206 (2.2)
Proximal humerus	2,355 (6.9)	276 (6.3)	528 (7.1)	167 (8.6)	1,017 (12.7)	4,343 (7.8)

Abbreviations: CI, confidence interval; IQR, interquartile range; SD, standard deviation.

^a^ Calculated for the patients experiencing a refracture only.

**FIGURE 1:**
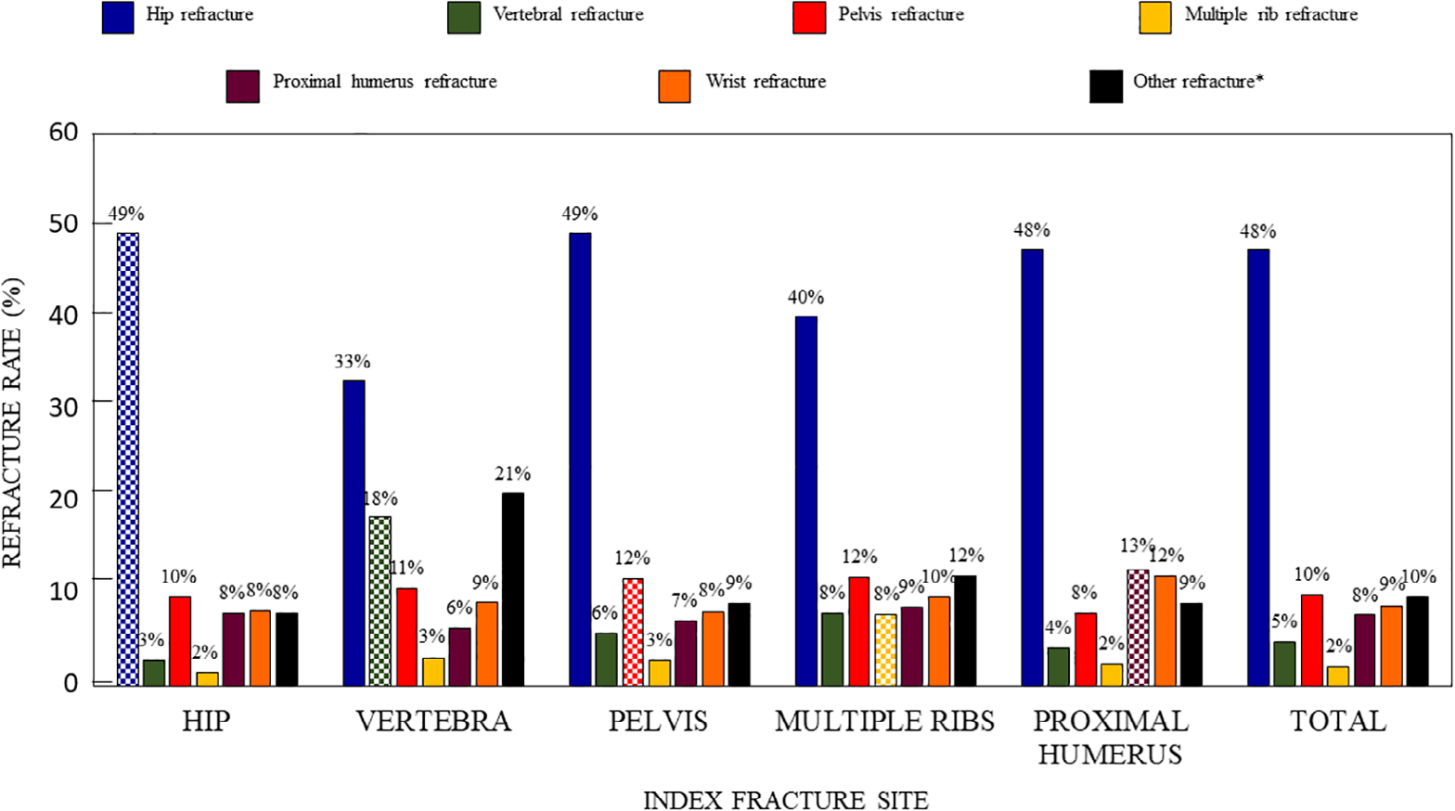
Refracture site according to index fracture site. The hatched columns indicate refractures at the same site as the index fracture. *Distal femur, tibia or forearm.

The most frequent refracture site was the hip, irrespective of the site of the index fracture, and these accounted for 47.7% of all refracture sites (Figure 1). In addition, refractures tended to occur more frequently at the same site as the index fracture rather than at a different site, notably for the hip, the pelvis, and vertebrae (Figure 1).

### 3.4 Mortality

During the follow‐up period, 138,286 patients died, of whom 8925 (2.5%) died during the index hospital stay. The mortality rate was highest for patients with index hip fractures, and these patients accounted for 83.1% of the deaths occurring during the index hospitalization (Table 4). In addition, patients with index hip fractures who died more rapidly after the index fracture compared to those with other index factures. Overall, the mortality rate at 12 months following the index fracture event was 12.8%. With respect to fracture site, 12‐month mortality rates were highest in patients with hip (16.6%) and pelvis (10.5%) fractures and lowest in patients with index vertebral fractures (5.0%) (Table 4). For the patients who died, the median survival time after the index fracture was 20.1 months. Kaplan‐Meier survival curves are presented by index fracture site in Supplemental Figure S2. The SMR in the year following the index fracture ranged from 1.66 for multiple rib fractures to 2.32 for hip fractures (Table 4).

**TABLE 4:** Mortality

	Site of index fracture					
Parameter	Hip (*n* = 208,102)	Vertebra (*n* = 31,979)	Pelvis (*n* = 38,051)	Multiple ribs (*n* = 17,184)	Proximal humerus (*n* = 52,468)	Total (*n* = 347,784)
Deaths, *n*	101,533	5798	13,902	4378	12,675	138,286
Mortality at 12 months, % (95% CI)	16.6 (16.4–16.7)	5.0 (4.7–5.2)	10.5 (10.2–10.8)	6.6 (6.2–6.9)	6.5 (6.3–6.7)	12.8 (12.7–12.9)
Mortality at 24 months, % (95% CI)	25.3 (25.2–25.5)	8.5 (8.2–8.8)	17.7 (17.3–18.1	11.7 (11.2–12.2)	11.0 (10.7–11.3)	20.1 (20.0–20.2)
Mortality at 36 months, % (95% CI)	33.9 (33.7–34.1)	12.0 (11.6–12.3)	25.0 (24.6–25.5)	16.5 (16.0–17.1)	15.6 (15.3–16.0)	27.3 (27.1–27.4)
Death during index stay, *n* (%)	7417 (3.4)	246 (0.8)	562 (1.5)	255 (1.5)	445 (0.8)	8925 (2.5)
Time from index fracture to death (month)^a^						
Median [IQR]	18.5 [4–38]	23.9 [8–43]	23.2 [8–42]	23.7 [8–43]	25.2 [9–45]	20.1 [5–40]
SMR (95%CI)	2.32 (2.29–2.34)	1.69 (1.61–1.78)	1.80 (1.74–1.86)	1.66 (1.56–1.76)	1.78 (1.72–1.84)	2.16 (2.14–2.18)

Abbreviations: CI, confidence interval; IQR, interquartile range; SMR, standardized mortality rate.

^a^ Calculated for the patients who died only.

**FIGURE 2:**
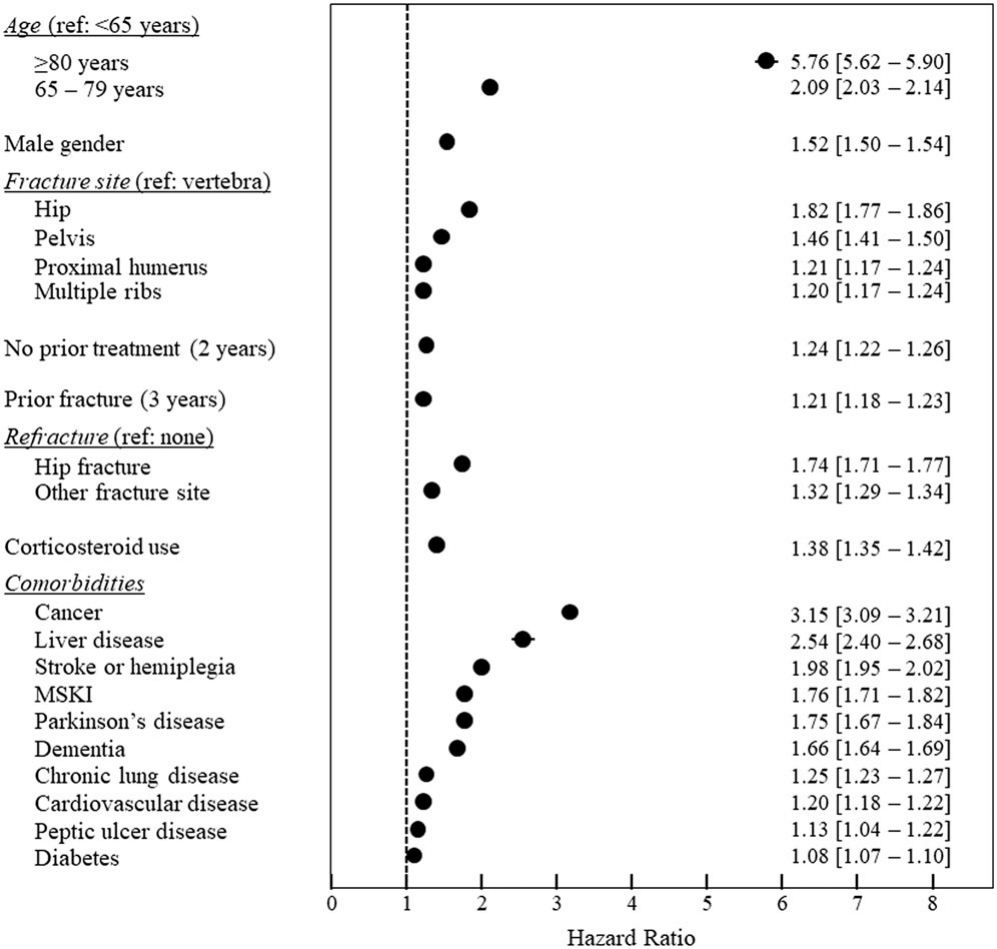
Risk factors for mortality (Cox model, multivariate analysis). Data are presented as hazard ratios with their 95% confidence intervals (in most cases these are within the diameter of the symbol). Refracture, corticosteroid therapy and comorbidities such as cancer, Parkinson's disease and stroke/hemiplegia were considered as time‐dependent variables. Abbreviation: MSKI, moderate or severe kidney injury.

A Cox analysis was performed to identify independent mortality risk factors. The results of the Cox analysis are presented in Figure 2. Apart from general mortality risk factors, such as older age, or the presence of life‐threatening comorbidities such as cancer, a number of osteoporosis‐related risk factors were identified. These include the site of the index fracture, with hip fractures being associated with the highest risk and vertebral fractures with the lowest risk, a previous osteoporotic fracture in the previous 3 years, refracture following the index fracture (in particular hip fracture), and no specific antiosteoporotic drug delivery in the 2 years prior to the index fracture.

## 4 DISCUSSION

The FRACTOS study demonstrates that, in a study population of over 350,000 eligible patients hospitalized for a severe osteoporotic fracture, the mortality risk is twofold to threefold higher than the refracture risk, although the two outcomes are not independent. At 12 months following a severe osteoporotic fracture, the mortality rate was 12.8% and the refracture rate 6.3%. For the patients who died, the median interval between the index fracture and death was only 20 months. The study also revealed that only 21% of all patients and 6% of treatment‐naïve patients were delivered a specific osteoporosis treatment within the year following the index fracture. Finally, a number of variables associated with postfracture mortality were identified, of which clinicians need to be aware in order to improve the standards of patient care.

Consistent with many previous studies,^(^ ^28^, ^29^, ^30^, ^31^ ^)^ we found that hip fractures were associated with a high mortality rate, accounting for three‐quarters of all deaths documented, with a 1‐year mortality rate of 17%. The SMR for hip fracture determined in this study was 2.32. This figure is somewhat lower than that reported from Australia a decade earlier in the Dubbo Osteoporosis Epidemiology Study (2.43 in women and 3.51 in men).^(^ ^4^ ^)^ In the Cox analysis, the relative mortality risk associated with hip fractures compared to vertebral fractures was 1.82.

In recent years, increased attention has been paid to the burden of non‐hip nonvertebral fractures.^(^ ^9^, ^32^, ^33^ ^)^ Pelvic fractures have also been associated with an elevated mortality in general population studies,^(^ ^34^ ^)^ patient registries,^(^ ^8^ ^)^ or relatively small cohort studies.^(^ ^8^, ^35^, ^36^, ^37^, ^38^ ^)^ In the present study, a group of over 38,000 hospitalized patients with pelvic fractures was followed. These patients presented a significantly increased mortality rate, second only to patients with hip fractures, with an SMR of 1.80 and a relative mortality risk compared to vertebral fractures of 1.46. In patients needing hospitalization for fractures, pelvic fractures thus have more severe consequences than vertebral fractures, although the latter have a higher visibility in osteoporosis research and practice guidelines. In particular, the rate of refracture at each time point studied was higher for pelvic fractures than for any other fracture location. Therapeutic studies, including trials of specific osteoporosis treatments, have not been conducted in patients with fractures of the pelvis, and more research is urgently needed in order to optimize therapeutic strategies in these patients.

Our study also identified an elevated mortality risk in patients hospitalized for multiple rib fractures, a class of osteoporotic fracture that has not been widely studied to date. Low‐trauma rib fracture is common in the elderly, with advanced age and osteoporosis being strong risk factors.^(^ ^39^ ^)^ Rib fractures, and even a single rib fracture, are associated with an increased risk of refracture and mortality.^(^ ^9^, ^40^, ^41^, ^42^ ^)^ In our study of 17,450 patients hospitalized with rib fractures, certain characteristics differ from those of other fracture types, in particular, as reported previously, a higher proportion of men (56.3%),^(^ ^9^ ^)^ as well as a slightly higher proportion of patients with chronic lung diseases, which has not, to our knowledge, been reported before. Refracture and mortality rates in these patients were comparable to those of patients with vertebral fractures. Our data, in the largest population of patients with rib fractures studied so far, have several implications, notably that, in spite of the fact that no clinical trials have been conducted in such patients, these patients should be considered for treatment.

Refracture rates differed markedly between index fracture sites, being highest for pelvic and hip fractures and lowest for multiple rib fractures. Although there was some tendency for fractures to occur at the same site as the index fracture, our study showed that, regardless of the index fracture location, there was one chance in two that the next fracture would be a hip fracture. For this reason, all patients with any severe fracture need to be managed carefully, regardless of the initial site, to prevent future hip fractures, which carry the highest burden of morbidity and mortality. Refracture events, and in particular hip refracture, were associated with increased mortality, as has been reported previously in the Dubbo Osteoporosis Epidemiology Study.^(^ ^43^ ^)^

This study also provides exhaustive information on specific antiosteoporosis treatments prescribed. Only 6.1% of previously treatment‐naïve patients hospitalized with severe fractures initiated such a treatment in the year following their index fracture, with a median delay of 6.3 months after the fracture. In spite of the benefits of antiosteoporotic drug treatment, the proportion of treated patients is thus very low. Even after taking into account patients previously treated prior to the index fracture, less than 21% of the patients received at least one prescription in the year following the fracture. These data suggest that the fracture event was not considered by healthcare professionals as an alert to initiate appropriate treatments for these patients. This demonstrates the failure of strategies for care of women experiencing osteoporotic fractures recommended in French^(^ ^21^ ^)^ and international^(^ ^20^ ^)^ guidelines, even in a country with universal access to reimbursed densitometry and treatments. However, the large proportion of patients receiving calcium and vitamin D suggest that the treatment gap is not due to lack of awareness by the physician of the osteoporotic disease underlying the fracture event, but rather to barriers to prescribing effective treatments that have been shown to reduce fracture risk. This finding should encourage the medical community to understand such barriers better, in order to improve the current paradigm of osteoporosis care.

The proportion of patients receiving a specific antiosteoporosis treatment after the fracture differs according to fracture site. Treatment rates in the year following the index fracture were highest for vertebral (25.8% of patients) and pelvis fractures (22.8%) and lowest for multiple rib fractures (10.3%). Only 15.1% of patients with hip fractures received a treatment after hip fracture, in spite of the fact that these patients have the highest mortality risk and that the efficacy of antiosteoporotic drug treatment after hip fractures in reducing this mortality has been demonstrated.^(^ ^44^ ^)^ It is possible that where the patient was hospitalized (medical or surgical unit) may influence whether the patient is directed to a fracture liaison service on discharge and thus on the probability of being prescribed a specific antiosteoporosis treatment.

Specific antiosteoporosis treatment was independently associated with lower mortality, an observation that has previously been made in a prospective cohort study.^(^ ^45^ ^)^ In our study, this effect may have been underestimated, because all patients with at least one delivery of medication were entered into the model, rather than those receiving a long‐term treatment. However, this finding should be interpreted with caution, because it may also be explained by residual confounding, despite multivariate adjustment. Bearing this in mind, these findings would encourage assessment of whether systematic evaluation of bone status during aging, and adapting treatment thereby, would not only reduce the risk of future fracture but also favor healthier and longer aging.

An important aspect of our study is the analysis of factors associated with an increased mortality risk. These include older age and certain potentially life‐threatening comorbidities, as well as fracture‐related variables such as fracture site and the occurrence of recurrent fractures. Our findings demonstrate that both patients with a history of prior fracture within the previous 3 years and patients with refracture have an increased risk of mortality, consistent with the notion that the more severe the disease, the higher the mortality rate observed.^(^ ^43^ ^)^ With respect to comorbidities, our study also highlights the impact of liver disease as a major risk factor for mortality following an osteoporotic fracture. Patients eligible for ALD status due to a chronic severe liver disease constitute 11.7% of the population with severe osteoporotic fractures. This observation is consistent with the findings of a recent large study of the Danish National Patient Registry, which also reported an increased mortality risk following hip fracture in patients with liver disease, and in particular cirrhosis,^(^ ^46^ ^)^ as well as those of a number of earlier studies using different sources and methodologies.^(^ ^47^, ^48^, ^49^ ^)^

The large size of the study sample also provided an opportunity to compare osteoporosis care between men and women. As previously demonstrated,^(^ ^50^ ^)^ the proportion of individuals with severe osteoporotic fractures was higher in women than in men. The distribution of fracture sites also differed, hip fractures being overrepresented in women and vertebral fractures overrepresented in men. Unexpectedly, men with severe osteoporotic fractures were on average younger than women; nonetheless, at least for hip fractures, the gender‐specific age distribution is very similar to that reported in the Danish National Hospital Discharge Register.^(^ ^51^ ^)^ Even after taking into account potential covariates in a Cox analysis, postfracture mortality was higher in men than in women, as reported in several previous studies.^(^ ^51^, ^52^, ^53^ ^)^ However, postfracture treatment rates were some fivefold lower in men. These findings emphasize the importance of recognizing and treating osteoporosis in men, who are at higher mortality risk. As is the case for fractures in general, the refracture rate was also lower in men. However, this result should be interpreted with caution, in the absence of analyses taking into account potential confounding factors, such as competing mortality or the distribution of fracture sites.

The FRACTOS study has several strengths and limitations. An important strength is the large sample size of a quasi‐exhaustive national database, covering 88% of the French population and including individuals of all social categories, regardless of gender and health status. Over 350,000 patients hospitalized for a fracture were included with a median duration of follow‐up of 39.1 months. This enables event rates to be estimated with precision and provides power to identify variables associated with mortality. A second strength is the possibility to describe several different types of severe osteoporotic fractures within the same population and database using the same definitions. Third, the data were collected in the context of monitoring healthcare resource consumption and not for research purposes, which should limit biases in data collection induced by the specific objective of the study. Although outcomes are relatively well documented for hip and vertebral fractures, the available literature on pelvic fractures and multiple rib fractures is much more limited. Limitations include a possible selection bias, as the diagnosis of osteoporosis, and the indication for treatment, could not be confirmed by densitometry, since the results of tests are not documented in the SNDS. Nonetheless, fragility fractures, which all eligible subjects presented, are a hallmark of osteoporosis, and severe fractures constitute an uncontested indication for antiosteoporotic treatment. Inclusion of patients who do not reach the threshold *T* score for osteoporosis may influence our mortality data, because excess mortality is observed mainly in subjects with low bone mineral density.^(^ ^11^ ^)^ Moreover, the study focuses only on patients who were hospitalized because of their fracture. Although we recognize that patients who were not hospitalized cannot be identified in the database, we anticipate that this would only concern a small number of patients with severe osteoporotic fractures. Another limitation is that the duration of the historical period prior fracture index was limited to 3 years, due to the availability of the database. For this reason, information on previous fracture and treatment history is restricted to the last two (treatments) or three (fractures) years prior to the index date and not all events may have been documented. Refractures are limited to hospitalized patients and thus some patients with wrist fractures, treated as outpatients, are missing from this prospective analysis. Certain individuals (12% of the French population) are covered by other insurance funds established for specific professions. A further 5% of patients with fractures were excluded because they were not insured continuously. However, there is no reason to think that the findings cannot be generalized to the entire French population, because in general population studies, osteoporotic fracture incidence in France has not been shown to differ according to professional status,^(^ ^54^, ^55^ ^)^ although it cannot be excluded that outcomes may differ somewhat.

In conclusion, this large national database study confirms the burden of severe osteoporotic fractures in terms of mortality risk, which is higher than the refracture risk. The study highlights the significant burden of certain non‐hip nonvertebral fractures, notably pelvis fractures, which have not been widely studied previously and contribute significantly to the burden of fragility fractures. We found no evidence for closing of the gap between those patients who deserve a treatment and who actually receive it. The findings of this study emphasize the crucial importance of better management of patients with severe fractures in order to improve survival as well as of developing effective strategies to reduce fracture risk in patients with osteoporosis. Meeting such goals would be expected to have significant benefits in terms of reduced morbidity and mortality.

## AUTHOR CONTRIBUTIONS

**Christian Roux:** Conceptualization; methodology; validation; writing‐review & editing. **Thierry Thomas:** Conceptualization; methodology; validation; writing‐review & editing. **Julien Paccou:** Conceptualization; methodology; validation; writing‐review & editing. **Geoffray Bizouard:** Conceptualization; data curation; formal analysis; methodology; project administration; writing‐review & editing. **Anne Crochard:** Conceptualization; funding acquisition; methodology; resources; supervision; validation; writing‐review & editing. **Emese Toth:** Supervision; validation; writing‐review & editing. **Magali Lemaitre:** Conceptualization; data curation; formal analysis; methodology; project administration; writing‐review & editing. **Frederique Maurel:** Conceptualization; data curation; formal analysis; methodology; project administration; writing‐review & editing. **Laure Perrin:** Conceptualization; funding acquisition; methodology; resources; supervision; validation; writing‐review & editing. **Florence Tubach:** Conceptualization; methodology; validation; writing‐review & editing.

## DISCLOSURES

Anne Crochard, Emese Toth, and Laure Perrin are employees of UCB Pharma SA. Geoffray Bizouard, Magali Lemaitre, and Frédérique Maurel are employees of IQVIA, a contract research agency responsible for the operational management of the study, funded by UCB Pharma SA. Christian Roux has received grants or honoraria from Alexion, Amgen, Regeneron and UCB. Thierry Thomas has received consultancy/speaker's fees from Amgen, Arrow, Biogen, Chugai, Expanscience, Grunenthal, Jansen, LCA, Lilly, MSD, Nordic, Novartis, Pfizer, Sanofi, Thuasne, Theramex, TEVA, and UCB, and financial support or fees for research activities from: Bone Therapeutics, Chugai, UCB; Julien Paccou has received honoraria from Amgen, MSD, Eli Lilly, Novartis, Pfizer, and UCB. Florence Tubach is head of the Centre de Pharmacoépidémiologie (Cephepi) of the Assistance Publique–Hôpitaux de Paris and of the Clinical Research Unit of Pitié‐Salpêtrière hospital; both these structures have received research funding and grants for the research projects handled and fees for consultancy activities from a large number of pharmaceutical companies, which have contributed indiscriminately to the salaries of its employees. Florence Tubach is not employed by these structures and has not receive any personal remuneration from these companies.

## AUTHOR CONTRIBUTIONS

The FRACTOS study was funded by UCB Pharma and Amgen. Anne Crochard, Emese Toth, and Laure Perrin initiated the study and contributed to the study design and interpretation of the findings. Geoffray Bizouard, Magali Lemaitre, and Frédérique Maurel were responsible for the operational management of the study and carried out the extraction of the SNDS database and the data analysis. Christian Roux, Thierry Thomas, Julien Paccou, and Florence Tubach constituted the study's scientific committee and oversaw the design of the study and interpretation of the findings. All authors contributed to the writing and finalization of the manuscript.

5

### PEER REVIEW

The peer review history for this article is available at https://publons.com/publon/10.1002/jbm4.10507.

## Supporting information


**Appendix S1**: Supporting information

## Data Availability

The study was performed in the SNDS database, which managed by the French national health insurance fund, the CNAMTS (*Caisse nationale de l'assurance maladie des travailleurs salariés*). Access to the database to institutions who meet the criteria for access to confidential data is permitted. Applications should be made to the National Health Data Institute (INDS; 19 rue Arthur Croquette, 94220 Charenton‐le‐Pont, Telephone: +33 1 45 18 43 90; Email: contact@indsante.fr; Website: https://www.indsante.fr/fr), which is responsible for access to all health data in France and is a one‐stop‐shop window for access to the SNDS database.
