# Supplementary material for: Refracture and mortality following hospitalization for severe osteoporotic fractures: The Fractos Study
Source: JBMR Plus. 2021 May 14;5(7):e10507. doi: 10.1002/jbm4.10507 (PMC8260818; doi:10.1002/jbm4.10507)
Supplement: Supplementary file 1 — Appendix S1: Supporting information [file JBM4-5-e10507-s001.docx]

# SUPPLEMENTAL MATERIAL

Supplemental Tables

Supplemental Table 1. Diagnostic codes for diagnosis of severe osteoporotic fractures

| INCLUSION CRITERIA | | | |
| --- | --- | --- | --- |
| **Fracture type** | **ICD-10** | **Description** |  |
| Hip | S720 | Fracture of neck of femur |  |
|  | S7200 | Fracture of neck of femur, closed |  |
|  | S721 | Pertrochanteric fracture |  |
|  | S7210 | Pertrochanteric fracture, closed |  |
|  | S722 | Sub-trochanteric fracture |  |
|  | S7220 | Sub-trochanteric fracture, closed |  |
| Vertebra | S220 | Fracture of thoracic vertebra |  |
|  | S2200 | Fracture of thoracic vertebra, closed |  |
|  | S320 | Fracture of lumbar vertebra d'une vertèbre lombaire |  |
|  | S3200 | Fracture of lumbar vertebra, closed |  |
| Pelvis | S321 | Fracture of sacrum |  |
|  | S3210 | Fracture of sacrum, closed |  |
|  | S322 | Fracture of coccyx |  |
|  | S3220 | Fracture of coccyx, closed |  |
|  | S323 | Fracture of ilium |  |
|  | S3230 | Fracture of ilium, closed |  |
|  | S324 | Fracture of acetabulum |  |
|  | S3240 | Fracture of acetabulum, closed |  |
|  | S325 | Fracture of pubis |  |
|  | S3250 | Fracture of pubis, closed |  |
|  | S327 | Multiple fractures of lumbar spine and pelvis |  |
|  | S3270 | Multiple fractures of lumbar spine and pelvis, closed |  |
|  | S328 | Fractures of other and unspecified parts of lumbar spine and pelvis |  |
|  | S3280 | Fractures of other and unspecified parts of lumbar spine and pelvis, closed |  |
| Multiple ribs | S224 | Multiple fractures of ribs |  |
|  | S2240 | Multiple fractures of ribs, closed |  |
| Proximal humerus | S422 | Fracture of upper end of humerus |  |
|  | S4220 | Fracture of upper end of humerus, closed |  |
| Osteoporosis with fracture | M80 | Osteoporosis with pathological fracture |  |

| EXCLUSION CRITERIA | | |
| --- | --- | --- |
| **Disease type** | **ICD-10** | **Description** |
| Paget’s disease | M88 | Paget's disease of bone [Osteitis deformans] |
| Tumours | C00-C97 | Malignant neoplasms |
| Infectious arthropathies | M00 | Pyogenic arthritis |
|  | M01 | Direct infections of joint in infectious and parasitic diseases classified elsewhere |
| Pathological fractures | M820 | Osteoporosis in multiple myelomatosis |
|  | M907 | Fracture of bone in neoplastic disease |
|  | M96 | Intraoperative and postprocedural complications and disorders of musculoskeletal system, not elsewhere classified |
| Traumatic lesions | T84 | Complications of internal orthopaedic prosthetic devices, implants and grafts |
|  | T912 | Sequelae of other fracture of thorax and pelvis |
|  | S797 | Multiple injury of hip and thigh |
|  | S798 | Other specified injuries of hip and thigh |
|  | S799 | Unspecified injury of hip and thigh |

Supplemental Table 2. Variables included in the Cox analysis

| Sociodemographic variables |
| --- |
| Age at index date |
| Gender |
| Osteoporosis-related variables |
| Site of index fracture |
| Previous fracture in two years before index date |
| Specific osteoporosis treatment in two years before index date |
| Comorbidities at index date |
| Chronic lung disease |
| Connective tissue disease |
| Cardiovascular disease |
| Peptic ulcer disease |
| Dementia |
| Liver disease |
| Diabetes |
| Moderate to severe kidney disease |
| HIV infection |
| Emerging medical conditions and treatments |
| Refracture |
| Stroke/hemiplegia |
| Cancer |
| Parkinson’s disease |
| Corticosteroid therapy |

**Supplemental Table 3. Characteristics of patients at the index hospitalisation by fracture type (analysis population: period 1st January 2009 – 31st December 2014) - by gender**

|  | **Hip** | **Vertebra** | **Pelvis** | **Multiple ribs** | **Proximal humerus** | **Total** |
| --- | --- | --- | --- | --- | --- | --- |
| **Patients (% of total)** | 215,672 (60.4%) | 32,231 (9.0%) | 38,620 (10.8%) | 17,450 (4.9%) | 52,922 (14.8%) | 356,895 (100%) |
| Men | 48 241 (47.0%) | 13 010 (14.3%) | 8 853 (9.7%) | 9 824 (10.8%) | 11 209 (12.3%) | 91 137 (100%) |
| Women | 167 431 (63.0%) | 19 221 (7.2%) | 29 767 (11.2%) | 7 626 (2.9%) | 41 713 (15.7%) | 265 758 (100%) |
| Gender (n, % women) | 167,431 (77.6%) | 19,221(59.6%) | 29,767 (77.1%) | 7,626 (43.7%) | 41,713 (78.8%) | 265,758 (74.5%) |
| **Fracture within 3 previous years** | 9 286 (4.3%) | 838 (2.6%) | 1 725 (19.5%) | 448 (2.6%) | 1 912 (3.4%) | 14 209 (4.0%) |
| Men | 1 247 (2.6%) | 194 (1.5%) | 170 (1.9%) | 114 (1.2%) | 278 (2.5%) | 2 003 (2.2%) |
| Women | 8 039 (4.8%) | 644 (3.3%) | 1 555 (5.2%) | 334 (4.4%) | 1 634 (3.9%) | 12 206 (4.6%) |
| **Age (years)** |  |  |  |  |  |  |
| Mean ± SD | 81.8 ± 10.6 | 70.5 ± 12.4 | 79.5 ± 11.8 | 71.9 ± 13.3 | 73.8 ± 12.1 | 78.8 ± 12.0 |
| Men | 76.9 ± 12.3 | 66.7 ± 11.6 | 71.7 ± 13.0 | 67.3 ± 12.1 | 67.7 ± 12.0 | 72.8 ± 13.0 |
| Women | 83.2 ± 9.6 | 73.0 ± 12.2 | 81.7 ± 10.3 | 77.9 ± 12.5 | 75.4 ± 11.6 | 80.9 ± 10.9 |
| ≤ 65 years | 20,623 (9.6%) | 12,042 (37.4%) | 5,779 (15.0%) | 6,255 (35.8%) | 14,309 (27.0%) | 59,008 (16.5%) |
| Men | 9 975 (20.7%) | 6 466 (49.7%) | 3 140 (35.5%) | 4 807 (48.9%) | 5 315 (47.4%) | 29 703 (32.6%) |
| Women | 10 648 (6.4%) | 5 576 (29.0%) | 2 639 (8.9%) | 1 448 (19.0%) | 8 994 (21.6%) | 29 305 (11.0%) |
| 65 – 80 years | 47,470 (22.0%) | 10,859 (33.7%) | 9,243 (23.9%) | 4,973 (28.5%) | 18,244 (34.5%) | 90,789 (25.4%) |
| Men | 13 528 (28.0%) | 4 200 (32.3%) | 2 686 (30.3%) | 2 993 (30.5%) | 3 591 (32.0%) | 26 998 (29.6%) |
| Women | 33 942 (20.3%) | 6 659 (34.6%) | 6 557 (22.0%) | 1 980 (26.0%) | 14 653 (35.1%) | 63 791 (24.0%) |
| >80 years | 147,579 (68.4%) | 9,330 (28.9%) | 23,598 (61.1%) | 6,222 (35.7%) | 20,369 (38.5%) | 207,098 (58.0%) |
| Men | 24 738 (51.3%) | 2 344 (18.0%) | 3 027 (34.2%) | 2 024 (20.6%) | 2 303 (20.5%) | 34 436 (37.8%) |
| Women | 122 841 (73.4%) | 6 986 (36.3%) | 20 571 (69.1%) | 4 198 (55.0%) | 18 066 (43.3%) | 172 662 (65.0% |
| **Charlson Score**  Mean ± SD | 0.6 ± 1.0 | 0.4 ± 0.9 | 0.5 ± 1.0 | 0.5 ± 1.0 | 0.5 ± 0.9 | 0.6 ± 1.0 |
| Men | 0.8 ± 1.3 | 0.5 ± 0.9 | 0.6 ±1.1 | 0.5 ± 1.0 | 0.5 ± 1.1 | 0.6 ± 1.2 |
| Women | 0.6 ± 1.0 | 0.4 ± 0.8 | 0.5 ± 1.0 | 0.6 ±1.0 | 0.6 ±1.0 | 0.5 ±0.9 |
| 0 | 130,917 (60.7%) | 22,730 (70.5%) | 24,809 (64.2%) | 11,183 (64.1%) | 35,242 (66.6%) | 224,881 (63.0%) |
| Men | 27 582 (57.2%) | 9 161 (70.4%) | 5 714 (64.5%) | 6 527 (66.4%) | 7 430 (66.3%) | 56 414 (61.9%) |
| Women | 103 335 (61.7%) | 13 569 (70.6%) | 19 095 (64.1%) | 4 656 (61.1%) | 27 812 (66.7%) | 168 467 (63.4%) |
| 1 – 2 | 74,021 (34.3%) | 8,609 (26.7%) | 12,199 (31.6%) | 5,565 (31.9%) | 16,035 (30.3%) | 116,429 (32.6%) |
| Men | 16 875 (35.0%) | 3 413 (26.2%) | 2 658 (30.0%) | 2 903 (29.6%) | 3 276 (29.2%) | 29 125 (32.0%) |
| Women | 57 146 (34.1%) | 5 196 (27.0%) | 9 541 (32.1%) | 2 662 (34.9%) | 12 759 (30.6% | 87 304 (32.9%) |
| 3 – 4 | 7,652 (3.5%) | 615 (1.9%) | 1,139 (2.9%) | 497 (2.8%) | 1,149 (2.2%) | 11,052 (3.1%) |
| Men | 2 519 (5.2%) | 285 (5.2%) | 319 (3.6%) | 271 (2.8%) | 325 (2.9%) | 3 719 (4.1%) |
| Women | 5133 (3.1%) | 330 (1.7%) | 820 (2.8%) | 226 (3.0%) | 824 (2.0%) | 7 333 (2.8%) |
| ≥5 | 3,082 (1.4%) | 277 (0.9%) | 473 (1.2%) | 205 (1.2%) | 496 (0.9%) | 4,533 (1.3%) |
| Men | 1 265 (2.6%) | 151 (1.2%) | 162 (1.8%) | 123 (1.3%) | 178 (1.6%) | 1 879 (2.1%) |
| Women | 1 817 (1.1%) | 126 (0.7%) | 311 (1%) | 82 (1.1%) | 318 (0.8%) | 2 654 (1.0%) |
| **Comorbidities^1^** |  |  |  |  |  |  |
| Diabetes | 23,950 (11.1%) | 3,700 (11.5%) | 4,532 (11.7%) | 2,411 (13.8%) | 7,547 (14.3%) | 42,140 (11.8%) |
| Men | 6 434 (13.3%) | 1 407 (10.8%) | 1 086 (12.3%) | 1 258 (12.8%) | 1 473 (13.1%) | 11 658 (12.8%) |
| Women | 18 184 (10.9%) | 2 143 (11.1%) | 3 416 (11.5%) | 1 089 (14.3%) | 6 020 (14.4%) | 30 852 (11.6%) |
| CLD | 24,452 (11.3%) | 3,766 (11.7%) | 4,695 (12.2%) | 2,699 (15.5%) | 6,092 (11.5%) | 41,704 (11.7%) |
| Men | 7 353 (15.2%) | 1 512 (11.6%) | 1 150 (13.0%) | 1 495 (15.2%) | 1 404 (12.5%) | 12 914 (14.2%) |
| Women | 17 099 (10.2%) | 2 254 (11.7%) | 3 545 (11.9%) | 1 204 (14.8%) | 4 688 (11.2%) | 28 790 (10.8%) |
| Dementia | 29,362 (13.6%) | 1,203 (3.7%) | 3,105 (8.0%) | 874 (5.0%) | 3,025 (5.7%) | 37,569 (10.5%) |
| Men | 4 982 (10.3%) | 331 (2.5%) | 439 (5.0%) | 273 (2.8%) | 364 (3.2%) | 6 389 (7.0%) |
| Women | 24 380 (14.6%) | 872 (4.5%) | 2 666 (9.0%) | 601 (7.9%) | 2 661 (6.4%) | 31 180 (11.7%) |
| Stroke | 7,432 (3.4%) | 595 (1.8%) | 948 (2.5%) | 386 (2.2%) | 1,052 (2.0%) | 10,413 (2.9%) |
| Men | 2 105 (4.4%) | 245 (1.9%) | 245 (2.8%) | 209 (2.1%) | 290 (2.6%) | 3 094 (3.4%) |
| Women | 5 327 (3.2%) | 350 (1.8%) | 703 (2.4%) | 177 (2.3%) | 762 (1.8%) | 7 319 (2.8%) |
| CHF | 9,825 (4.6%) | 697 (2.2%) | 1,583 (4.1%) | 568 (3.3%) | 1,121 (2.1%) | 13,794 (3.9%) |
| Men | 2 713 (5.6%) | 275 (2.1%) | 374 (4.2%) | 259 (2.6%) | 294 (2.6%) | 3 915 (4.3%) |
| Women | 7 112 (4.2%) | 422 (2.2%) | 1 209 (4.1%) | 309 (4.1%) | 827 (2.0%) | 9 879 (3.7%) |
| MI | 3,494 (1.6%) | 309 (1.0%) | 521 (1.3%) | 227 (1.3%) | 486 (0.9%) | 5,037 (1.4%) |
| Men | 1 252 (2.6%) | 181 (1.4%) | 166 (1.9%) | 136 (1.4%) | 169 (1.5%) | 1 904 (2.1%) |
| Women | 2 242 (1.3%) | 128 (0.7%) | 355 (1.2%) | 91 (1.2%) | 317 (0.8%) | 3 133 (1.2%) |

^1^Only comorbidities used to construct the Charlson comorbidity index and identified in >1% of patients overall are listed. CLD: chronic lung disease; CHF: congestive heart failure; MI: myocardial infarction.

**Supplemental Table 4. Specific antiosteoporotic drug treatments - by gender**

|  | **Hip** | **Vertebra** | **Pelvis** | **Multiple ribs** | **Proximal humerus** | **Total** |
| --- | --- | --- | --- | --- | --- | --- |
| **Follow-up population** | **N = 208,102** | **N = 31,979** | **N = 38,051** | **N = 17,184** | **N = 52,468** | **N = 347,784** |
| Men | n = 45 646 | n = 12 882 | n = 8 639 | N = 9 679 | n = 11 705 | n = 87 941 |
| Women | n = 167 431 | n = 19 221 | n = 29 767 | N = 7 626 | n = 41 713 | n = 265 758 |
| *Before index fracture* |  |  |  |  |  |  |
| **At least one delivery** | **32,930 (15.8%)** | **6,125 (19.2%)** | **9,270 (24.4%)** | **2,200 (12.8%)** | **8,761 (16.7%)** | **59,286 (17.0%)** |
| Men | 1 489 (3.3%) | 441 (3.4%) | 338 (3.9%) | 240 (2.5%) | 290 (2.6%) | 2 798 (3.2%) |
| Women | 31 441 (19.4%) | 5 684 (29.8%) | 8 932 (30.4%) | 1 960 (26.1%) | 8 471 (20.5%) | 56 488 (21.7%) |
| ***At time of index fracture*** | 15,273 (7.3%) | 3,390 (10.6%) | 5,056 (13.3%) | 1,203 (7.0%) | 4,450 (8.5%) | 29,372 (8.4%) |
| Men | 1 963 (4.3%) | 982 (7.6%) | 337 (3.9%) | 198 (2.0%) | 320 (2.9%) | 3 800 (4.3%) |
| Women | 16 003 (9.9%) | 4 178 (21.9%) | 3 300 (11.2%) | 560 (7.5%) | 4 496 (10.9%) | 28 537 (11.0%) |
| *During 12 mo after index fracture* | | | | | | |
| **At least one delivery** | 31,385 (15.1%) | 8,250 (25.8%) | 8,683 (22.8%) | 1,775 (10.3%) | 8,127 (15.5%) | 58,220, (16.7%) |
| Men | 2 020 (4.4%) | 1 060 (8.2%) | 428 (5.0%) | 244 (2.5%) | 318 (2.9%) | 4 080 (4.6%) |
| Women | 29 365 (18.1%) | 7 190 (37.6%) | 8 255 (28.1%) | 1 531 (20.4%) | 6 572 (15.9%) | 54 140 (20.8%) |
| **Treatment continued** | 15,273 (7.3%) | 3,390 (10.6%) | 5,056 (13.3%) | 1,203 (7.0%) | 4,450 (8.5%) | 29,372 (8.4%) |
| Men | 667 (1.5%) | 275 (2.1%) | 195 (2.3%) | 124 (1.3%) | 132 (1.2%) | 1 393 (1.6%) |
| Women | 14 606 (9.0%) | 3 115 (16.3%) | 4 861 (16.5%) | 1 079 (14.4%) | 4 318 (10.4%) | 27 979 (10.8%) |
| **Treatment restarted** | 4,048 (1.9%) | 1,112 (3.5%) | 1,166 (3.1%) | 196 (1.1%) | 1,098 (2.1%) | 7,620 (2.2%) |
| Men | 121 (0.3%) | 46 (0.4%) | 19 (0.2%) | 20 (0.2%) | 36 (0.3%) | 242 (0.3%) |
| Women | 3 927 (2.4%) | 1 066 (5.6%) | 1 147 (3.9%) | 176 (3.9%) | 1 062 (2.6%) | 7 378 (2.8%) |
| **Treatment initiated** | 12,064 (5.8%) | 3,748 (11.7%) | 2,461 (6.5%) | 376 (2.2%) | 2,579 (4.9%) | 21,228 (6.1%) |
| Men | 1 232 (2.7%) | 739 (5.7%) | 214 (2.5%) | 100 (1.0%) | 160 (1.4%) | 2 445 (2.8%) |
| Women | 10 832 (6.7%) | 3 009 (15.8%) | 2 247 (7.6%) | 276 (3.7%) | 2 419 (5.8%) | 18 783 (7.2%) |

Treatment continued: treatment ongoing at time of index fracture and delivery continuing without interruption thereafter.

Treatment restarted: delivery of a previous treatment after the index fracture, following a period of interruption.

Treatment initiation (no treatment in the two years preceding the index fracture and first delivery documented after the index fracture.

**Supplemental Table 5. Refracture during follow up after index fracture – by gender**

|  | **Site of index fracture** | | | | | |  |
| --- | --- | --- | --- | --- | --- | --- | --- |
|  | **Hip** | **Vertebra** | **Pelvis** | **Multiple ribs** | **Proximal humerus** | **Total** | |
| **Follow-up population (N, % of total)** | **208 102 (59.8%)** | **31 979 (9.2%)** | **38051 (10.9%)** | **17 184 (4.9%)** | **52 468 (15.1%)** | **347 784 (100%)** | |
| *Men* | *45 646 (51.9%)* | *12 882 (14.7%)* | *8 639 (9.7%)* | *9 679 (11.0%)* | *11 075 (12.6%)* | *87 921 (100%)* | |
| *Women* | *162 456 (62.5%)* | *19 097 (7.3%)* | *29 412 (11.3%)* | *7 505 (2.9%)* | *41 393 (15.9%)* | *259 863 (100%)* | |
| **Refracture following fracture (N,%)** | **34 039 (16.4%)** | **4 372 (13.7%)** | **7 440 (19.6%)** | **1 948 (11.3%)** | **8 032 (15.3%)** | **55 831 (16.1%)** | |
| *Men* | *5 372 (11.7%)* | *1 096 (8.35%)* | *941 (10.9%)* | *664 (6.9%)* | *1 178 (10.6%)* | *9251 (10.2%)* | |
| *Women* | *28 667 (17.6%)* | *3 276 (17.2%)* | *6 499 (22.1%)* | *1 284 (17.1%)* | *6 854 (16.6%* | *46 580 (17.9%)* | |
| **Refracture rate at 12 mo** | **6.6% [6.5%-6.7%]** | **5.5% [5.3%- 5.8%]** | **7.8% [7.5%- 8.1%]** | **4.0% [3.7%- 4.3%]** | **5.1% [4.9%-5.3%]** | **6.3% [6.2%-6.3%]** | |
| *Men* | *5.3% (5.1%-5.5%)* | *4.1% (3.7%-4.4%)* | *5.2% (4.8%-5.7%)* | *2.2% (1.9%-2.5%)* | *3.7% (3.3%-4.0%)* | *4.5% [4.4%-4.6%]* | |
| *Women* | *7.0% (6.9%-7.1%)* | *6.5% (6.1%-6.9%)* | *8.5% (8.2%-8.8%)* | *6.2% (5.7%-6.8%)* | *5.4% (5.2%-5.7%)* | *6.8% [6.7%-6.9%]* | |
| **Refracture rate at 24 mo** | **11.7% [11.6%-11.9%]** | **9.0% [8.8%-9.3%]** | **13.3% [12.9%-12.7%]** | **7.1% [6.7%-7.5%]** | **9.0% [8.8%-9.3%]** | **10.9% [10.8%-11.1%]** | |
| *Men* | *9.2% (8.9%-9.5%)* | *6.2% (5.8%-6.7%)* | *8.0% (7.4%-8.6%)* | *4.1% (3.7%-4.6%)* | *6.5% (6.0%-7.0%)* | *7.6% [7.4%-7.4%]* | |
| *Women* | *12.4% (12.2%-12.6%)* | *10.8% (10.3%-11.3%)* | *14.8% (14.4%-15.3%)* | *11.0% (10.2%-11.7%)* | *9.7% (9.4%-10.0%)* | *12.0% [11.9%-12.2%]* | |
| **Refracture rate at 36 mo** | **16.1% [15.9%-16.3%]** | **11.6% [11.2%-11.9%]** | **18.0% [17.6%-18.4%]** | **9.6% [9.1%-10.0%]** | **12.5% [12.2%-12.8%]** | **14.9% [14.7%-15.0%]** | |
| *Men* | *12.8% (12.4%-13.1%)* | *7.7% (7.2%-8.2%)* | *10.5% (9.8%-11.3%)* | *5.8% (5.3%-6.3%)* | *9.1% (8.5%-9.7%)* | *10.3% [10.1%-10.6%]* | |
| *Women* | *16.9% (16.7%-17.1%)* | *14.1% (13.6%-14.6%)* | *20.1% (19.6%-20.6%)* | *14.5% (13.7%-15.4%)* | *13.3% (13.0%-13.7%)* | *16.3% [16.2%-16.5%]* | |
| **Time from index fracture to refracture (mo)** | | | | | | |  |
| **Mean ± SD** | **23.7 ± 19.6** | **23.0 ± 20.1** | **23.0 ± 19.5** | **24.8 ± 20.1** | **26.1 ± 20.6** | **24.0 ± 19.8** | |
| *Men* | *22.5 ±19.1* | *20.1 ±19.5* | *20.2 ±19.1* | *25.1 ±19.4* | *25.6 ±20.3* | *22.6 ± 19.4* | |
| *Women* | *24.0 ±19.7* | *24 ±20.3* | *23.4 ±19.5* | *24.6 ±20.5* | *26.2 ±20.7* | *24.2 ±19.9* | |
| **Median [IQR]** | **18.7 [8 – 35]** | **17.4 [6 – 35]** | **18.0 [7 – 34]** | **20.0 [8 – 37]** | **21.5 [9 – 39]** | **19.0 [8 – 36]** | |
| *Men* | *17.6 [6.7 – 33.5]* | *13.6 [4.6 – 29.8]* | *14.6 [4.5 – 30.5]* | *20.6 [9.1 – 38.1]* | *21.3 [8.6 – 37.7]* | *17.6 [6.5 – 33.7]* | |
| *Women* | *19.0 [7.8 – 35.5]* | *18.6 [7 – 36.2]* | *18.4 [7.1 – 34.7]* | *19.7 [7.6 – 36.9]* | *21.5 [9.1 – 39.2]* | *19.3 [7.9 – 35.9]* | |
| **Site of first refracture** | | | | | | |  |
| **Hip** | **16,794 (49.3%)** | **1,453 (33.2%)** | **3,661 (49.2%)** | **783 (40.2%)** | **3,814 (47.5%)** | **26,505 (47.5%)** | |
| *Men* | *2 848 (53.0%)* | *192 (3.6%)* | *415 (7.7%)* | *136 (2.5%)* | *372 (6.9%)* | *4 429 (47.9%)* | |
| *Women* | *13 946 (48.6%)* | *758 (2.6%)* | *2 890 (10.1%)* | *383 (1.3%)* | *1 983 (6.9%)* | *22 076 (47.4%)* | |
| **Vertebra** | **950 (2.8%)** | **804 (18.4%)** | **429 (5.8%)** | **155 (8.0%)** | **341 (4.2%)** | **2,679 (4.8%)** | |
| *Men* | *338 (30.8%)* | *294 (26.8%)* | *135 (12.3%)* | *66 (6.0%)* | *64 (5.8%)* | *749 (8.1%)* | |
| *Women* | *1 115 (34.0%)* | *510 (15.6%)* | *330 (10.1%)* | *68 (2.1%)* | *212 (6.5%)* | *1 930 (4.1%)* | |
| **Pelvis** | **3,305 (9.7%)** | **465 (10.6%)** | **871 (11.7%)** | **232 (11.9%)** | **638 (7.9%)** | **5,511 (9.9%)** | |
| *Men* | *426 (45.3%)* | *111 (11.8%)* | *120 (12.8%)* | *56 (6.0%)* | *58 (6.2%)* | *807 (8.7%)* | |
| *Women* | *3 235 (49.8%)* | *318 (4.9%)* | *751 (11.6%)* | *150 (2.3%)* | *470 (7.2%)* | *4 704 (10.1%)* | |
| **Multiple ribs** | **519 (1.5%)** | **134 (3.1%)** | **206 (2.8%)** | **151 (7.8%)** | **196 (2.4%)** | **1,206 (2.2%)** | |
| *Men* | *246 (37.0%)* | *82 (12.3%)* | *65 (9.8%)* | *93 (14.0%)* | *57 (8.6%)* | *404 (4.4%)* | |
| *Women* | *537 (41.8%)* | *73 (5.7%)* | *167 (13.0%)* | *58 (4.5%)* | *110 (8.6%)* | *802 (1.7%)* | |
| **Proximal humerus** | **2,355 (6.9%)** | **276 (6.3%)** | **528 (7.1%)** | **167 (8.6%)** | **1,017 (12.7%)** | **4,343 (7.8%)** | |
| *Men* | *571 (48.5%)* | *70 (5.9%)* | *72 (6.1%)* | *53 (4.5%)* | *194 (16.5%)* | *745 (8.1%)* | |
| *Women* | *3 243 (47.3%)* | *271 (4.0%)* | *566 (8.3%)* | *143 (2.1%)* | *823 (12.0%)* | *3 598 (7.7%)* | |

CI: confidence interval; IQR: interquartile range; SD: standard deviation.

**Supplemental Table 6. Mortality during follow up after index fracture – by gender**

| **Site of index fracture** | | | | | | | | | | | |
| --- | --- | --- | --- | --- | --- | --- | --- | --- | --- | --- | --- |
|  | **Hip** | **Vertebra** | | **Pelvis** | | **Multiple ribs** | | **Proximal humerus** | | **Total** | |
|  | N = 208,102 | N = 31,979 | | N = 38,051 | | N = 17,184 | | N = 52,468 | | N = 347,784 | |
| Deaths (N) | 101,533 | 5,798 | | 13,902 | | 4,378 | | 12,675 | | 138,286 | |
| Mortality at 12 mo (%) | 16.6 [16.4 – 16.7] | 5.0 [4.7 – 5.2] | | 10.5 [10.2 – 10.8] | | 6.6 [6.2 – 6.9] | | 6.5 [6.3 – 6.7] | | 12.8 [12.7 – 12.9] | |
| Men | 21.2 [20.8 – 21.6] | 5.3 [5.0 – 5.8] | | 11.0 [10.4 – 11.7] | | 5.3 [4.9 – 5.8] | | 8.1 [7.6 – 8.6] | | 14.4 [14.2 – 14.6] | |
| Women | 15.3 [15.1 – 15.4] | 4.7 [4.4 – 5.0] | | 10.3 [10.0 – 10.7] | | 8.1 [7.5 – 8.8] | | 6.0 [5.8 – 6.3] | | 12.2 [12.1 – 12.4] | |
| Mortality at 24 mo (%) | 25.3 [25.2 – 25.5] | 8.5 [8.2 – 8.8] | | 17.7 [17.3 – 18.1 | | 11.7 [11.2 – 12.2] | | 11.0 [10.7 – 11.3] | | 20.1 [20.0 – 20.2] | |
| Men | 30.5 [30.1 – 30.9] | 8.6 [8.1 – 9.1] | | 17.0 [16.2 – 17.9] | | 9.8 [9.2 – 10.4] | | 13.4 [12.7 – 14.0] | | 21.4 [21.2 – 21.7] | |
| Women | 23.9 [23.7 – 24.1] | 8.5 [8.1 – 8.9] | | 17.9 [17.4 – 18.3] | | 14.1 [13.3 – 14.9] | | 10.4 [10.1 – 10.7] | | 19.6 [19.5 – 19.8] | |
| Mortality at 36 mo (%) | 33.9 [33.7 – 34.1] | 12.0 [11.6 – 12.3] | | 25.0 [24.6 – 25.5] | | 16.5 [16.0 – 17.1] | | 15.6 [15.3 – 16.0] | | 27.3 [27.1 – 27.4] | |
| Men | 38.9 [38.4 – 39.4] | 11.6 [11.1 – 12.2] | | 22.6 [21.7 – 23.6] | | 13.4 [12.8 – 14.2] | | 18.1 [17.4 – 18.9] | | 27.8 [27.5 – 28.1] | |
| Women | 32.5 [32.3 – 32.8] | 12.2 [11.7 – 12.7] | | 22.7 [21.8 – 23.6] | | 20.4 [19.5 – 21.4] | | 15.0 [14.6 – 15.3] | | 27.1 [26.9 – 27.3] | |
| Death during index stay | 7,417 (3.4%) | 246 (0.8%) | | 562 (1.5%) | | 255 (1.5%) | | 445 (0.8%) | | 8,925 (2.5%) | |
| Men | 2 500 (10.5%) | 124 (5.8%) | | 210 (7.9%) | | 136 (6.9%) | | 129 (4.6%) | | 3 099 (9.3%) | |
| Women | 4 917 (6.3%) | 122 (3.3%) | | 352 (3.1%) | | 119 (4.9%) | | 316 (3.2%) | | 5 826 (5.6%) | |
| Time from index fracture to death (mo) | | |  | |  | |  | |  | |  |
| Median [IQR] (mo) [IQR] | 18.5 [4 – 38] | 23.9 [8 – 43] | | 23.2 [8 – 42] | | 23.7 [8 – 43] | | 25.2 [9 – 45] | | 20.1 [5 – 40] | |
| Men | 11.9 [2 – 31] | 19.9 [6 – 39] | | 15.8 [4 – 34] | | 21.7 [7 – 41] | | 21.3 [6 – 40] | | 14.2 [3 – 33] | |
| Women | 20.5 [5 – 40] | 26.4 [10 – 46] | | 24.8 [9 – 43] | | 25.5 [9 – 44] | | 26.5 [10 – 46] | | 21.9 [6 – 41] | |
| SMR [95%CI] | 2.32 [2.29 – 2.34] | 1.69 [1.61 – 1.78] | | 1.80 [1.74 – 1.86] | | 1.66 [1.56 – 1.76] | | 1.78 [1.72 – 1.84] | | 2.16 [2.14 – 2.18] | |
| Men | 3.01 [2.95 – 3.07] | 1.95 [1.80 – 2.10] | | 2.36 [2.21 – 2.52] | | 1.73 [1.58 – 1.88] | | 2.57 [2.40 – 2.74] | | 2.75 [2.71 – 2.80] | |
| Women | 2.13 [2.10 – 2.16] | 1.54 [1.44 – 1.64] | | 1.67 [1.62 – 1.74] | | 1.61 [1.48 – 1.74] | | 1.60 [1.54 – 1.67] | | 1.99 [1.97 – 2.01] | |

CI: confidence interval; IQR: interquartile range; SD: standard deviation; SMR: standardised mortality rate.

**Supplemental Figures - Legends**

**Supplemental Figure 1.** Patient flow-chart

^1^History of Paget’s disease, cancer, infectious arthritis, or bone fragility secondary to malignant disease or to surgical interventions.

^2^Covered by more than one Health Insurance Regimen during the study period.

Percentages are calculated with respect to the preceding line.

Supplemental Figure 2. Survival analysis of refracture and mortality

Type of index fracture: blue: hip; yellow: vertebra; red: pelvis; green: multiple ribs; black: proximal humerus.

The numbers under the curves indicate the number of subjects at risk at each time point.

**Supplemental Figures**

**Supplemental Figure 1**. Patient flow-chart

Supplemental Figure 2. Survival analysis of refracture and mortality

| **A. Refracture: all sites** | **B. Refracture: by site** |
| --- | --- |
| 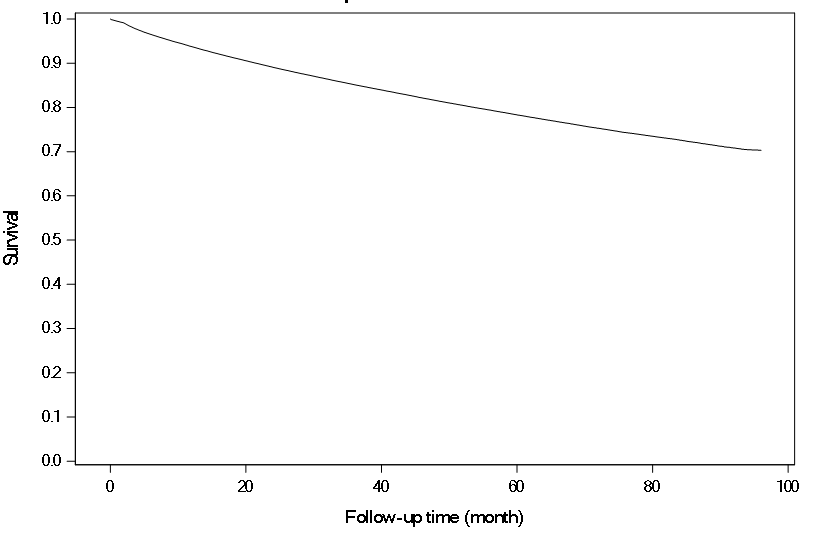 |  |
| **C. Mortality: all sites** | **D. Mortality: by site** |
| 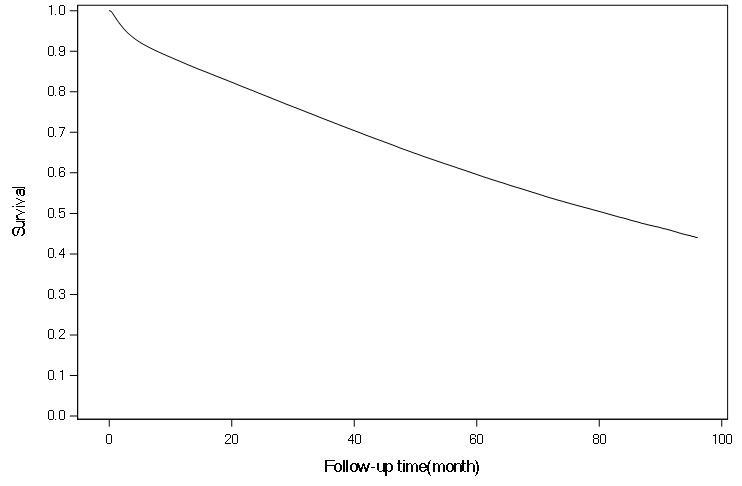 |  |
